# Enhanced Biomass and Protein Synthesis in Engineered *Cyberlindnera jadinii* Growing on Ethanol/Acetate: Metabolic Engineering and Transcriptomic Mechanism

**DOI:** 10.3390/foods15091464

**Published:** 2026-04-22

**Authors:** Yixin Cao, Longxue Ma, Yaxiang Li, Zhen Zhu, Yu Duan, Wenqin Bai, Liucheng Long, Pengbao Shi, Limei Chen, Demao Li

**Affiliations:** 1 College of Food Science & Technology, Hebei Normal University of Science & Technology, Qinhuangdao 066004, China; 2 Tianjin Key Laboratory for Industrial Biological System and Bioprocessing Engineering, Tianjin Institute of Industrial Biotechnology, Chinese Academy of Sciences, Tianjin 300308, China

**Keywords:** *Cyberlindnera jadinii*, fermentation optimization, acylating acetaldehyde dehydrogenase, transcriptomic analysis, ethanol, acetate

## Abstract

Producing single-cell protein (SCP) from syngas-derived ethanol and acetate offers a sustainable solution to global protein shortages, yet microbial utilization mechanisms for these mixtures remain underexplored. This study establishes a systematic bioconversion strategy using *Cyberlindnera jadinii* TU389. To mitigate acetaldehyde accumulation during ethanol metabolism, we engineered the strain TU546 to overexpress acylating acetaldehyde dehydrogenase (ADA6). TU546 achieved a maximum biomass of 46.7 g/L and a protein yield of 21.69 g/L, representing enhancements of 28.16% and 23.02% over the wild-type, respectively. Transcriptomic analysis revealed extensive metabolic reprogramming. In the C2 assimilation pathway, upregulated aldehyde dehydrogenase and acetyl-CoA Synthetase 1 accelerated acetate conversion to acetyl-CoA, while downregulated pyruvate decarboxylase and alcohol dehydrogenase minimized carbon flux loss. The upregulation of tricarboxylic acid cycle enzymes, the glyoxylate shunt, and acyl-coA oxidase improved carbon skeleton retention. Moreover, the upregulation of transaminases and N-acetylglutamate synthase, synergized with intensified cell proliferation signaling, redirected amino acid metabolism toward a synthesis-enhanced and degradation-controlled paradigm. This synergistic regulatory network drives the high-efficiency bioconversion of ethanol and acetate into SCP, establishing a molecular mechanistic foundation for the valorization of syngas-derived C2 substrates in biological macromolecule production.

## 1. Introduction

As the fundamental components of living organisms, proteins are of paramount importance across the food, animal feed, and pharmaceutical sectors [1,2]. At present, however, a critical protein supply–demand gap is emerging globally. Propelled by persistent population growth and shifting dietary preferences, global protein demand is forecast to increase by 30–50% by 2050 [3,4]. Meanwhile, the capacity of traditional livestock and aquaculture systems is increasingly restricted by intensive resource consumption, environmental degradation, and frequent disease outbreaks [5,6]. This intensifying crisis has highlighted the urgent requirement for more sustainable protein production paradigms. Consequently, single-cell protein (SCP) derived from microbial biosynthesis has gained significant traction as a high-potential solution. By bypassing the need for arable land and offering significantly shorter production cycles, SCP represents a highly efficient pathway that also holds substantial promise for advancing carbon neutrality.

Utilizing C1 source gases for microbial protein biosynthesis constitutes a green and sustainable approach that facilitates carbon cycling and minimizes environmental impact. Syngas (primarily composed of CO, CO_2_, and H_2_), derived from the gasification of industrial off-gases or municipal solid waste, serves as a crucial C1 feedstock. In recent years, microbial syngas fermentation has gained significant traction as a primary means of producing clean energy [7]. Through the Wood-Ljungdahl pathway in microorganisms such as *Clostridium autoethanogenum*, syngas is efficiently converted into ethanol and acetate mixtures [8], This biochemical process unlocks significant potential for generating non-food, low-carbon feedstocks, thereby providing a renewable foundation for modern biomanufacturing [9,10,11]. For instance, Beijing Shougang LanzaTech Technology Co., Ltd. has successfully commercialized the use of *Clostridium* to valorize industrial tail gases, effectively turning waste into valuable resources. However, the excessively high costs of traditional separation processes for this C2-containing liquid mixture severely limit its industrial economic viability [12]. In addition, reports on high-performance engineered strains capable of synthesizing microbial protein from C2 substrates are still scarce. Consequently, there is an urgent need to develop downstream bioprocesses that can directly and efficiently utilize this C2 mixture to achieve effective conversion in the C1-C2-SCP process.

*Cyberlindnera jadinii* (anamorph: *Candida utilis*), an industrial microorganism Generally Recognized as Safe (GRAS), exhibits unique application value [13]. Supported by comprehensive genetic and phenotypic insights [14], this strain exhibits a broad metabolic spectrum and is capable of utilizing a wide range of carbon sources, such as ethanol, acetate, xylose, and various agro-industrial residues for sustainable SCP production [15,16]. This implies that the strain harbors a highly developed and intricate metabolic network. Characterized by a high microbial protein content (32–75%), a balanced amino acid profile, and an abundance of bioactive compounds, *C. jadinii* has received both international and domestic regulatory approvals, paving the way for its application in the food and feed sectors [17]. Studies have demonstrated that *C. jadinii* possesses health-enhancing effects in livestock and that it has been formulated as a commercial feed supplement [18,19]. In recent years, gene-editing technologies, notably CRISPR-Cas9, have surmounted the genetic manipulation challenges posed by its polyploid genome [20,21,22,23]. This advancement has enabled the successful engineering of the strain into cell factories for producing high-value-added products, including L-lactic acid, isopropanol, biotin, cholesterol, and heterologous steroids [23,24,25,26], demonstrating its immense potential as a synthetic biology chassis through multidimensional metabolic engineering [27].

Despite its significant advantages, the application of wild-type *C. jadinii* in industrial fermentation is yet to be fully exploited due to existing constraints. A primary challenge during ethanol-based fermentation is the rapid accumulation of toxic intermediates, most notably acetaldehyde, which severely compromises biomass accumulation. An effective strategy to resolve this issue is to mitigate the acetaldehyde-induced inhibition of the strain through enhancing its ability to utilize acetaldehyde. Accumulating evidence has shown that overexpression of the acetaldehyde dehydrogenase (ALDH) gene mitigates acetaldehyde toxicity, and a naturally occurring ADA6 (can convert acetaldehyde to acetyl-CoA) variant with enhanced catalytic efficiency has been systematically screened and characterized, exhibiting approximately a 14.1-fold increase in activity relative to the wild-type control [28]. As a critical metabolic node, ALDH catalyzes the transformation of acetaldehyde into either acetate or acetyl-CoA, the latter of which serves as an indispensable precursor for the biosynthesis of various high-value metabolites [29,30,31,32,33].

The primary objective of this research is to construct a resilient biomanufacturing platform for C2 valorization, utilizing *C. jadinii* for the high-efficiency conversion of ethanol and acetate into high-value SCP. To further augment protein productivity, a dual strategy involving bioprocess optimization and metabolic engineering was implemented to circumvent growth inhibition caused by acetaldehyde stress. This proposed C2-SCP pathway provides a novel framework for the effective utilization of C2 intermediates derived from the bioconversion of C1-based syngas. By improving the overall economic competitiveness of gas-to-protein technologies, this work provides critical technical and theoretical support for the large-scale industrialization of the integrated C1–C2–SCP production chain.

## 2. Materials and Methods

### 2.1. Strain Screening

A 10 g barley sample was shaken thoroughly in 0.7% sterile normal saline, and the supernatant was collected. Subsequently, 1 mL supernatant was inoculated into 100 mL of liquid YM medium (3 g/L yeast extract, 3 g/L malt extract, 5 g/L peptone, 10 g/L glucose) and incubated at 28 °C with shaking at 180 rpm for 24 h. The culture was then streaked onto the surface of solid YM medium (supplemented with 20 g/L agar) using an inoculating loop following the four-quadrant streaking method. The streaked plates were inverted and incubated at 28 °C for 48 h.

### 2.2. Shake-Flask Fermentation Optimization

Initially, five ethanol-to-acetate mass ratios (1:1, 2:1, 3:1, 1:2, and 1:3), at a total carbon source concentration of 10 g/L, were evaluated to determine the optimal carbon source ratio, with biomass, protein content, and protein yield used as comprehensive evaluation indices. Subsequently, comparative single-factor experiments were carried out for inorganic nutrients, including KH_2_PO_4_ (1–3 g/L), MgSO_4_ (0–2 g/L), CaCl_2_ (0–2 g/L), FeSO_4_·7H_2_O (0–50 mg/L), and ZnSO_4_·7H_2_O (0–0.2 g/L). Finally, the initial pH (4.0–9.0) and fermentation temperature (25–37 °C) were further optimized separately based on the optimized medium. While the initial fermentations were conducted at 28 °C with an unadjusted natural pH (referencing standard yeast cultivation protocols), this systematic optimization definitively established 31 °C and an initial pH of 7.0 as the final optimal conditions for the strain TU389.

### 2.3. Scale-Up Bioreactor Fermentation

*C. jadinii* was cultivated in a 5 L bioreactor with 3.5 L of optimized medium. Logarithmic-phase seeds were inoculated at 10% (*v*/*v*). Fed-batch cultivation utilized an ethanol/acetate mixture as the carbon source at 31 °C, with an agitation of 200–500 rpm and aeration at 1 vvm. Feeding was triggered when dissolved oxygen (DO) exceeded 30%. A two-stage feeding strategy was employed: manual supplementation (5 g/L) from 0 to 12 h, followed by DO-stat automatic feeding (0.5–1.5 g/min) to maintain DO at 30%. Temperature, pH, DO, and OD_600_ were monitored online, with periodic sampling for metabolite quantification and growth monitoring.

### 2.4. Determination of Biomass, Protein Content, Protein Yield, and Conversion Yield

Methods were adapted from Tong et al. [34]. Briefly, 30 mL of culture broth was centrifuged (10,000 rpm, 4 °C, 10 min), washed with sterile water, and lyophilized for 48 h to a constant weight. Biomass (g/L) was calculated as follows:

Biomass (g/L) = (M1 − M0)/0.03

where M0 and M1 represent the weights (g) of the empty and cell-containing tubes, respectively.

For protein analysis, lyophilized cells were pulverized and sieved (100-mesh). The nitrogen content of a 3.2–3.8 mg sample was determined using a Flash smart elemental analyzer (Thermo Ltd., Waltham, MA, USA). Protein content and yield were calculated using a conversion factor of 6.25:

Protein content (%) = Nitrogen content × 6.25 (protein conversion factor)

Protein yield (g/L) = Biomass × Protein content

The conversion yield (carbon source to biomass) was determined as

Conversion yield (%) = [Biomass (g DCW/L)/Consumed carbon source concentration (g/L)] × 100%

### 2.5. Determination of Strain Susceptibility

The YM seed culture of *C. jadinii TU389* was cultivated in a shake flask at 28 °C with shaking at 180 rpm until the OD_600_ reached 1.0. Subsequently, 100 μL aliquot of the seed culture was spread onto YM agar plates containing varying concentrations of Geneticin (G418, 0–250 mg/L) or Hygromycin B (HygB, 0–250 mg/L). The inoculated plates were incubated at 28 °C under constant temperature conditions for 2–3 d to evaluate the resistance tolerance of *C. jadinii* TU389 to G418 and HygB.

### 2.6. Construction of Gene Overexpression Vectors

Plasmids were constructed using pMD18 as the backbone. Linearization was performed using specific restriction enzymes (e.g., BamHI, EcoRI, SmaI, or ApaI) followed by purification. Base plasmid pMD18-Hyg was generated by assembling a promoter, a HygB, and a CYC1 terminator. Subsequently, four autonomous replication sequences (ARS1-4) were inserted to create replicon plasmids, with pMD18-Hyg-ARS4 selected for further engineering. A beta-glucuronidase (GUS) reporter was fused to HygB via a linker to form pMD18-Hyg-ARS4-GUS. Finally, the acylating acetaldehyde dehydrogenase gene (*ada6*) was placed under the control of either the P_GAPDH_ or P_PGK_ promoter using seamless cloning technology (homologous arms). All heterologous sequences were codon-optimized for *C. jadinii* and synthesized (GENEWIZ, Suzhou, China), while endogenous elements were PCR-amplified from *C. jadinii* TU389 genomic DNA. Strains, plasmids, and primers are detailed in Tables S1–S4.

### 2.7. Transformation and Verification of Recombinant Strains

Plasmid transformation was conducted as per Gu et al. [23]. Transformants were verified by GUS histochemical staining and evaluated for expression stability. Single colonies were subcultured onto yeast malt (YM) agar containing 450 μg/mL HygB. Colonies (1–2 mm) were treated with 10 μL GUS staining solution (Beyotime Biotechnology, Shanghai, China) and incubated at 37 °C for 1–2 h in the dark. GUS-positive clones (blue coloration) were then subcultured for 3–5 successive generations on HygB-selective plates. GUS staining at each passage confirmed stable plasmid expression under selection pressure prior to subsequent experiments.

### 2.8. Chemical Quantification of Fermentation Metabolites

Ethanol and acetate concentrations in the fermentation broth were quantified using high-performance liquid chromatography (HPLC), as previously described [35]. For the microbial protein evaluation, the amino acid profile of the lyophilized *C. jadinii* biomass was determined by a certified third-party analytical testing agency (SGS-CSTC Standards Technical Services Co., Ltd., Qingdao, China). To ensure the accurate quantification of all amino acids, three distinct hydrolysis procedures were strictly employed according to standard analytical protocols. For the determination of the majority of amino acids, samples were hydrolyzed with 6 M HCl at 110 °C for 24 h. Because sulfur-containing amino acids are susceptible to degradation, cysteine and methionine were quantified after pre-oxidizing the samples with performic acid, followed by standard acid hydrolysis. Additionally, tryptophan, which is completely destroyed by acid hydrolysis, was determined independently following alkaline hydrolysis using NaOH. The final amino acid contents were expressed as grams per 100 g of total protein (g/100 g).

### 2.9. Transcriptome Sequencing and Analysis

TU546 cells (cultured at pH 6.0 and 5.5 in a 5 L bioreactor) and TU389 cells (control, pH 6.0) were harvested, snap-frozen in liquid nitrogen, and dispatched to Shanghai Majorbio Bio-pharm Technology Co., Ltd., Shanghai, China, for RNA sequencing (RNA-seq). Raw reads were filtered to remove adapters, low-quality sequences, and reads with high proportions of unknown bases (N) to obtain high-quality clean reads. Subsequent bioinformatics processing and data visualization were conducted on the Majorbio Cloud Platform.

### 2.10. Data Processing and Statistical Analysis

All experiments were performed in triplicate (n = 3). Data were analyzed using IBM SPSS Statistics 25 (IBM Corp., Armonk, NY, USA). After confirming normality and homogeneity of variance, significant differences between groups were determined via one-way analysis of variance (ANOVA). Lowercase letters in figures denote statistical significance (*p* < 0.05), where shared letters indicate no significant difference (*p* > 0.05). Graphical representations were generated using Origin 2025 (OriginLab, Northampton, MA, USA).

## 3. Results

### 3.1. Screening and Identification of C. jadinii

**Figure 1:**
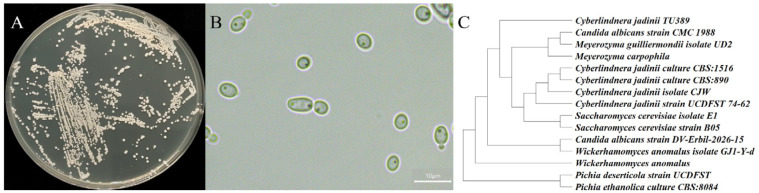
Morphology and phylogenetic studies of the strain TU389: (**A**) colony on plate; (**B**) cell morphology under microscope; (**C**) phylogenetic tree.

A non-model yeast strain was successfully isolated from barley samples via systematic screening and isolation protocols. On YM solid medium, the colonies presented a creamy white phenotype, characterized by a smooth and moist surface and regular edges (Figure 1A,B). Phylogenetic analysis was performed based on the partial sequences of the ITS1 and ITS4 regions, and the phylogenetic tree (constructed using the Maximum Likelihood method in MEGA 11 software) was compared with closely related species retrieved from the NCBI GenBank database (Figure 1C). While the strain showed evolutionary homology with *Saccharomyces* and certain *Pichia* species, it shared the highest sequence identity (99.6%) and phylogenetic similarity with most strains identified as *C. jadinii*. Therefore, the strain was identified as a novel isolate of *C. jadinii* and named a TU389.

### 3.2. Fermentation Optimization

Single-factor optimization was conducted at the shake-flask scale to optimize key process parameters. With respect to the carbon source, a mixed substrate consisting of ethanol and acetate ratio of 2:1(total carbon concentration of 10 g/L) was employed, yielding a biomass of 3.45 ± 0.02 g/L and a protein yield of 1.37 ± 0.06 g/L. This specific ratio was chosen because it lies within the representative range of ethanol/acetate ratios generated during syngas fermentation by *Clostridium* species in laboratory studies, which ensures the industrial applicability of the substrate formulation. Optimization of inorganic salt components revealed the optimal concentrations as follows: 1.5 g/L KH_2_PO_4_, 0.5 g/L CaCl_2_, 1.0 g/L MgSO_4_, 30 mg/L FeSO_4_·7H_2_O, and 0.1 g/L ZnSO_4_·7H_2_O. Importantly, Zn^2+^ exhibited a pronounced promotional effect on protein synthesis, elevating the protein yield to 2.28 ± 0.04 g/L [36]. This observation is in line with previous reports on *Fusarium venenatum*, where the addition of 20 mg/L ZnSO_4_·7H_2_O enhanced protein production by 120% [34], indicating a conserved regulatory role of Zn^2+^ in microbial protein synthesis.

**Figure 2:**
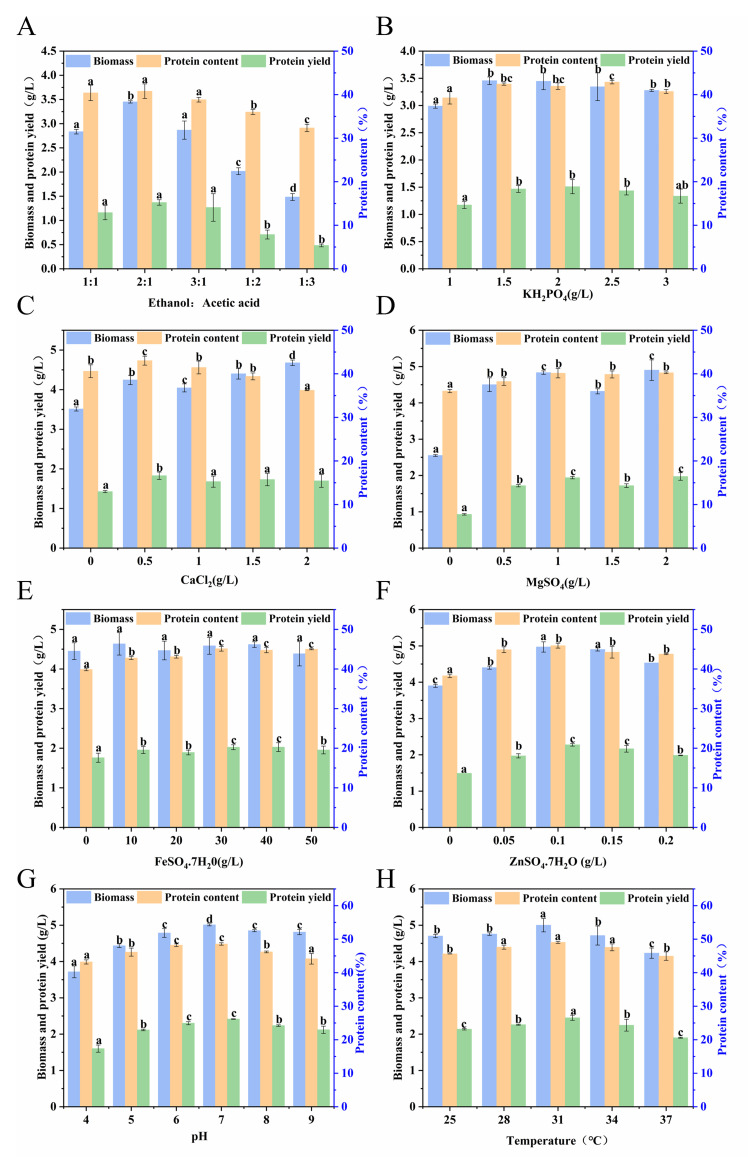
Optimization of shake flask fermentation for the strain TU389: (**A**) optimization of carbon source ratio; (**B**) optimization of KH_2_PO_4_; (**C**) optimization of CaCl_2_; (**D**) optimization of MgSO_4_; (**E**) optimization of FeSO_4_·7H_2_O; (**F**) optimization of ZnSO_4_·7H_2_O; (**G**) optimization of pH; (**H**) optimization of temperature.Different lowercase letters (a, b, c.) above the bars indicate significant differences (*p* < 0.05) among different treatments. The same letter represents no significant difference.

The optimal initial pH and culture temperature were determined to be 7.0 and 31 °C, respectively. Under these integrated optimized conditions (optimal ethanol-to-acetate ratio, inorganic salt concentrations, initial pH, and temperature), the maximum biomass attained 5.0 ± 0.18 g/L, with a peak protein yield of 2.45 ± 0.07 g/L (Figure 2A–H). Collectively, the systematic optimization led to a 44.9% increase in biomass (from 3.45 ± 0.02 g/L to 5.0 ± 0.18 g/L), a 15.7% improvement in protein content (from 42.4% to 49.05%), and a 78.8% elevation in protein yield (from 1.37 ± 0.06 g/L to 2.45 ± 0.07 g/L). Additionally, the carbon-to-biomass conversion yield was significantly improved from 34% to 50%, indicating enhanced carbon source utilization efficiency.

**Figure 3:**
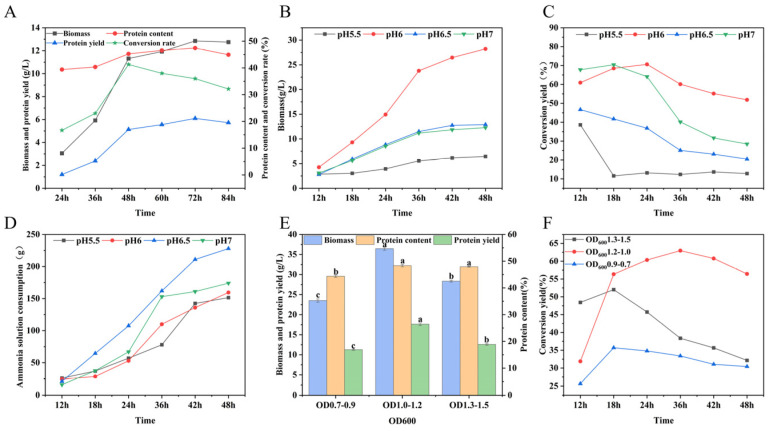
Optimization of 5 L fermenter culture of the strain TU389. (**A**) Biomass, protein content and protein yield at pH 7. (**B**) Biomass at different pH conditions. (**C**) Conversion yield at different pH conditions. (**D**) Ammonia consumption at different pH conditions. (**E**) Biomass, protein content and protein yield at pH 6 with different inoculation OD_600_. Different lowercase letters (a, b, c.) above the bars indicate significant differences (*p* < 0.05) among different treatments. The same letter represents no significant difference.(**F**) Conversion yield at pH 6 with different inoculation OD_600_.

Shake-flask cultivation of *C. jadinii* often suffers from metabolic disturbances due to unstable pH. In a 5 L bioreactor, biomass and protein yield peaked at 48 h (Figure 3A), which was identified as the optimal harvest time; beyond this point, substrate conversion efficiency declined. pH significantly regulated substrate assimilation. Maintaining a constant pH 6.0 facilitated acetate entry into the tricarboxylic acid (TCA) cycle, yielding a maximum biomass of 28.22 ± 0.76 g/L, a protein yield of 13.97 ± 0.33 g/L, and a 51.85% conversion yield (Figure 3B–D). This inoculum density was further optimized at pH 6.0 (Figure 3E,F). An initial OD_600_ of 1.0–1.2 significantly outperformed other densities, achieving a peak biomass of 36.44 ± 0.39 g/L, a protein content of 48.38 ± 0.12%, and a protein yield of 17.63 ± 0.34 g/L. In contrast, higher or lower inoculation densities resulted in markedly lower biomass (23.51–28.34 g/L). In conclusion, a high-efficiency SCP production system was established using a DO-stat feeding strategy (30% DO) at pH 6.0, effectively scaling the process from laboratory flasks to a 5 L bioreactor.

### 3.3. Metabolic Engineering of the Ethanol Utilization Pathway Reduces the Acetaldehyde Toxicity

#### 3.3.1. Antibiotic Susceptibility Testing

To establish a transformation system for *C. jadinii* TU389, susceptibility to hygromycin B (Hyg B) and geneticin (G418) was evaluated. The minimum inhibitory concentration (MIC) for G418 was 200 μg/mL, while Hyg B effectively inhibited wild-type growth at 150 μg/mL (Figure S1A), consistent with previous reports [37]. However, electroporation significantly enhanced antibiotic tolerance in *C. jadinii*, a phenomenon likely driven by membrane-stress responses or genomic perturbations common in yeast species [38,39,40,41]. Consequently, the initial MIC was insufficient to eliminate false positives post-pulsing. Through gradient optimization (Figure S1B), 450 μg/mL Hyg B was identified as the optimal concentration to completely suppress the growth of electroporated wild-type cells while ensuring the robust selection of positive transformants. This optimized selective pressure provided a reliable foundation for subsequent genetic engineering.

#### 3.3.2. Overexpression of Acylating Acetaldehyde Dehydrogenase ADA6

**Figure 4:**
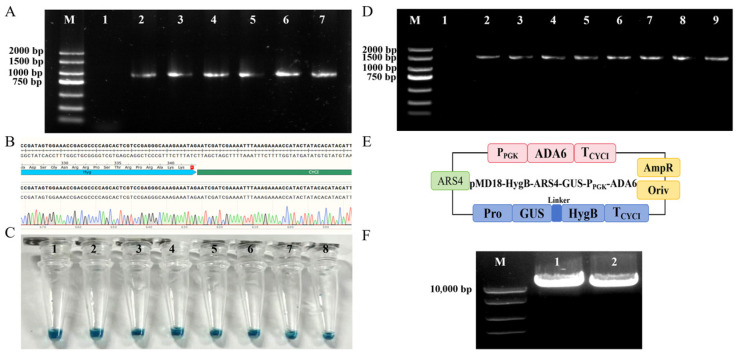
Validation process of plasmid construction. (**A**) Electrophoretogram of PCR verification for the HygB fragment of the Pro-Hyg-CYC1 expression cassette: M: DNA Marker; Lane 1: Wild-type the strain TU389 control; Lanes 2–7: Positive transformant clones. (**B**) Sequencing results of the ligation of HygB and CYC1 terminator. (**C**) GUS staining verification of transformants: 1–4: staining results of P_GAPDH_-ADA6 transformants; 5–8: staining results of P_PGK_-ADA6 transformants. (**D**) Colony PCR verification results of ADA6 promoter P_GAPDH_ and P_PGK_ transformants: M: DNA Marker; Lane 1: Wild-type control; Lanes 2–5: Colony verification results of P_GAPDH_-ADA6 transformants; Lanes 6–9: Colony verification results of P_PGK_-ADA6 transformants. (**E**) Maps of recombinant plasmids pMD18-HygB-ARS4-GUS-P_PGK_-ADA6. (**F**) Electrophoretogram of plasmid restriction enzyme digestion verification: Lane 1–2: pMD18-HygB-ARS4-GUS-P_PGK_-ADA6 plasmid digested with ApaI.

Based on the aforementioned screening system, core genetic manipulation elements specifically suited for TU389 were further developed and validated. Initially, the Pro-Hyg-CYC1 expression cassette was randomly integrated into the genome via electroporation. Successful transformation and stable inheritance were confirmed through colony PCR, sequencing, and stability testing over 10 consecutive generations, demonstrating the feasibility of the fundamental transformation protocol (Figure 4A,B). Subsequently, the functional performance of four candidate autonomously replicating sequences (ARSs) was evaluated by monitoring plasmid retention rate under antibiotic selection pressure. It was found that only the recombinant plasmid harboring ARS4 maintained stable inheritance for more than 12 generations. Consequently, ARS4 was selected as the essential component for the backbone plasmid in subsequent manipulations. To validate fusion expression functionality, a GUS-HygB fusion expression plasmid containing a flexible linker sequence (Gly-Gly-Ser-Gly) was successfully constructed. GUS staining confirmed the effective co-expression of both genes. Notably, in the absence of selective pressure, the plasmid remained stable for 5 generations but was completely cured (naturally lost) by the 6th generation (Figure S2), demonstrating its significant potential for application in markerless genome editing [42]. Finally, through antibiotic screening and GUS staining, two endogenous promoters, P_GAPDH_ (glyceraldehyde-3-phosphate dehydrogenase promoter) and P_PGK_ (phosphoglycerate kinase promoter), were identified for their stable performance during continuous subculturing (Figure 4C–E). These results provide reliable transcriptional regulatory elements for subsequent metabolic engineering studies.

**Figure 5:**
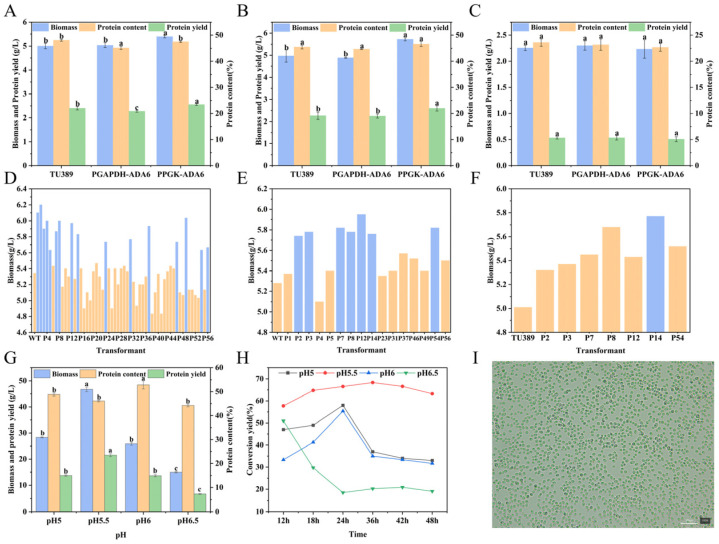
Fermentation characteristics of P_GAPDH_-ADA6 and P_PGK_-ADA6 transformants under different carbon sources, transformant screening results, and optimization in a 5 L fermenter: (**A**) growth after 48 h fermentation with ethanol/acetate mixed carbon source; (**B**) growth after 48 h fermentation with ethanol as the sole carbon source; (**C**) growth after 48 h fermentation with acetate as the sole carbon source; (**D**–**F**) evaluation results of the first, second, and third rounds of transformant screening, respectively, where the blue bars represent the superior transformants; (**G**) biomass, protein content, and protein yield of the strain TU546 under different pH conditions; (**H**) total conversion yield of the strain TU546 at different time points under different pH conditions; (**I**) morphology of the strain TU546 after 48 h fermentation in a 5 L fermenter at pH 5.5.Different lowercase letters (a, b, c.) above the bars indicate significant differences (*p* < 0.05) among different treatments. The same letter represents no significant difference.

To construct an engineered *C. jadinii* strain capable of efficiently utilizing the mixed ethanol/acetate carbon source, systematic promoter screening, stable genetic integration, and scale-up fermentation validation were conducted. Initially, the expression efficiencies of the *ada6* gene driven by the P_GAPDH_ and P_PGK_ promoters were compared. Shake-flask fermentations (Figure 5A–C) demonstrated that under both mixed carbon source and sole ethanol conditions, the biomass and protein yield of the P_PGK_ transformants were significantly superior to those of the wild-type and P_GAPDH_ transformants. However, no significant differences were observed under the sole acetate condition. This observation perfectly aligns with the molecular mechanism that acetaldehyde dehydrogenase is not the primary rate-limiting enzyme in acetate metabolism [43], thereby confirming P_PGK_ as the optimal strong promoter for this study.

To obtain an engineered strain with a stable genetic background suitable for process scale-up, the plasmid containing the P_PGK_-ADA6 expression cassette was linearized (Figure 4F) to facilitate random genomic integration via the yeast non-homologous end joining (NHEJ) mechanism [44]. The 56 transformants, designated P1-P56, were maintained through continuous subculturing under antibiotic selection. To verify the successful integration and stable inheritance of the exogenous *ada6* gene, GUS staining was performed at each generation. Following three rounds of rigorous shake-flask fermentation screening (Figure 5D–F), the best-performing engineered strain, P14, was isolated. This strain achieved a biomass of 5.77 g/L on the mixed carbon source and was formally designated as *C. jadinii* TU546.

#### 3.3.3. Evaluation of the Protein Production of *C. jadinii* TU546 in a 5 L Bioreactor

Scale-up validation was performed in a 5 L fermenter using a simulated syngas-derived ethanol/acetate mixture. Using the wild-type optimal pH (6.0) as control, four pH gradients (5.0, 5.5, 6.0, and 6.5) were established to systematically investigate their effects on TU546 fermentation. The results showed that although biomass reached its maximum at 48 h under all conditions, key performance indicators differed greatly (Figure 5G,H). The strain TU546 achieved its optimal performance at pH 5.5, reaching a biomass of 46.7 ± 0.90 g/L and a protein content of 46.44 ± 0.36%, which corresponded to a total protein yield of 21.69 ± 0.56 g/L. The representative cell morphology under these optimal conditions is illustrated in Figure 5I. This indicates a strong balance between cell growth and protein synthesis. Although the highest protein content (52.83 ± 1.68%) was obtained at pH 6.0, the low biomass (25.87 ± 0.62 g/L) led to a lower protein yield (13.66 ± 0.40 g/L). Cell growth was inhibited at pH 6.5, with only 14.99 ± 0.22 g/L biomass, while pH 5.0 produced 28.32 ± 0.20 g/L biomass.

Conversion yield analysis further supported these results (Figure 5H). The pH 5.5 group reached a maximum conversion yield of 68.33% at 36 h and remained high at 63.23% until 48 h, performing significantly better than all other groups. In contrast, the conversion yield in the other groups decreased continuously and markedly: by 48 h, the pH 5.0 and pH 6.0 groups dropped to 33.43% and 31.66%, respectively. The sharpest decrease occurred at pH 6.5, where the conversion yield declined steadily after 12 h and fell to only 19.04% at the end of fermentation. This trend was consistent with the poor biomass accumulation at this pH. Taken together, these results demonstrate that pH 5.5 is the optimal condition for achieving efficient substrate-to-biomass conversion from C2 substrates in *C. jadinii* TU546.

### 3.4. The Amino Acid Composition of C. jadinii *TU546*

This study further determined the amino acid composition of TU546 under steady-state fermentation conditions at pH 5.5 in a 5 L bioreactor. The results revealed a total amino acid content of 48.79 g/100 g biomass, indicating a high overall amino acid abundance and a comprehensive profile within the microbial protein of the strain. As shown in Table 1, glutamic acid, aspartic acid, leucine, and lysine were the most abundant amino acids, whereas tryptophan and cystine exhibited the lowest levels. Overall, this profile represents the typical amino acid distribution characteristic of microbial proteins. From an industrial application perspective, the essential amino acids leucine and lysine both reached considerable levels in TU546. Coupled with the phenotype of high biomass accumulation, this indicates that the pH 5.5 condition not only facilitates efficient biomass generation but also yields a microbial protein with an essential amino acid profile highly suitable for animal feed or industrial protein applications.

### 3.5. Transcriptomic Analysis of the Phenotypic Differentiation Mechanisms in C. jadinii *TU546* and *TU389*

#### 3.5.1. Transcriptome Sequencing and Differential Gene Expression Profiling

**Figure 6:**
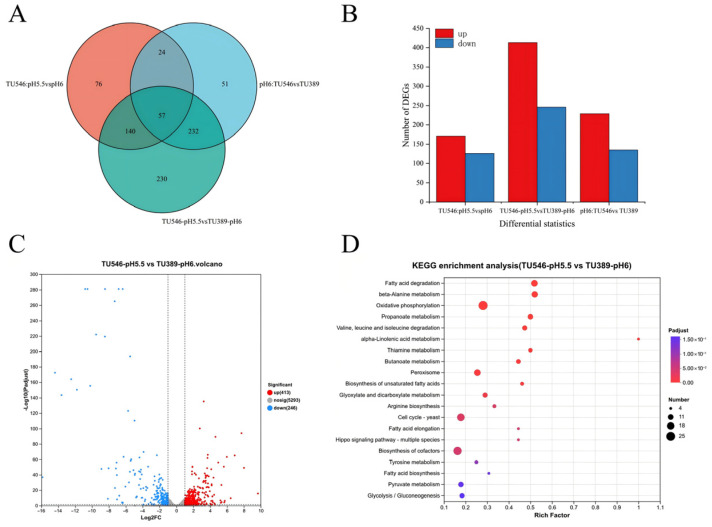
Characterization of Differentially Expressed Genes in Transcriptome: (**A**) Venn diagram of differentially expressed genes; (**B**) numbers of up-regulated and down-regulated genes in different groups; (**C**) volcano plot of differentially expressed genes between TU546-pH5.5 and TU389-pH6; (**D**) bubble plot of KEGG enrichment analysis for TU546-pH5.5 vs. TU389-pH6.

Transcriptome sequencing of the nine samples generated 59.36 Gb of clean data. Each individual sample yielded over 5.79 Gb, with Q30 scores exceeding 95.88% (Figures S4 and S5 and Table S5), demonstrating high data reliability. A Venn diagram analysis identified 57 cores differentially expressed genes (DEGs) shared across all comparison groups (Figure 6A). The comparison between *C. jadinii* TU546 (pH 5.5) and TU389 (pH 6.0) exhibited the highest transcriptomic variance, identifying 659 DEGs (413 upregulated and 246 downregulated). This was followed by 364 DEGs in the TU546 (pH 6.0) vs. TU389 (pH 6.0) group, and 297 DEGs in the acid-stress group (TU546 at pH 5.5 vs. pH 6.0) (Figure 6B).

Gene Ontology (GO) and KEGG enrichment analyses (Figure 6C,D) revealed that under optimal pH conditions, DEGs were primarily associated with catalytic and transporter activities, as well as lipid metabolism. Specifically, key genes involved in glycolysis, oxidative phosphorylation, and fatty acid degradation were significantly upregulated in TU546. Under acidic pressure (pH 5.5 vs. 6.0), TU546 showed enrichment in pathways related to transmembrane transport, nucleotide metabolism, and cofactor biosynthesis. Furthermore, at pH 6.0, TU546 exhibited prioritized degradation of fatty acids and branched-chain amino acids (BCAAs) compared to the wild-type strain.

#### 3.5.2. Transcriptional Alterations of Carbon Metabolism-Related Genes

**Figure 7:**
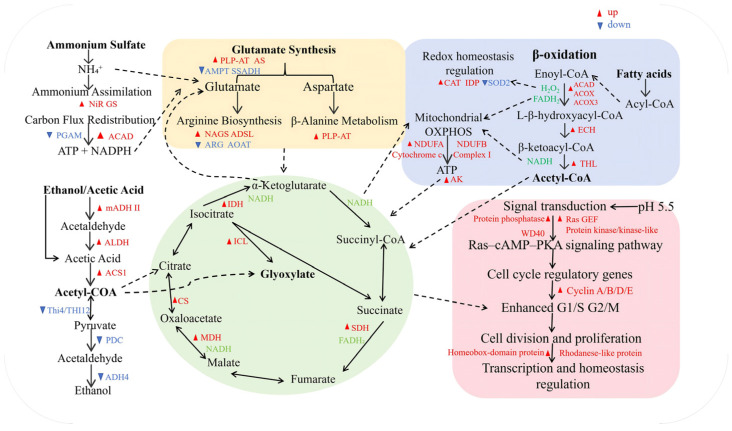
Metabolic pathway map of TU546 under pH 5.5 condition.

Transcriptomic profiling revealed a significant shift in carbon metabolism in TU546 at pH 5.5. Genes including mADH II, ALDH, and ACS were synchronously upregulated, while PDC (log_2_FC = −2.26) and ADH4 were downregulated (Figure 7 and Table S6). Concurrently, genes encoding citrate synthase (CS), malate dehydrogenase (MDH), and isocitrate lyase (ICL) were significantly upregulated. The expression of adenylate kinase (AK) also increased at pH 5.5, while thiamine metabolism genes (THI4, THI12) were downregulated. Phenotypically, TU546 at pH 5.5 achieved a higher biomass (46.7 vs. 25.87 g/L) and carbon conversion yield (63.23% vs. 31.66%) compared to the pH 6.0 condition.

#### 3.5.3. Transcriptional Changes in Lipid Catabolism and Energy Metabolism Pathways

At pH 5.5, key β-oxidation enzymes (ACAD, ACOX, and THL) were significantly upregulated in TU546, alongside an increase in peroxisome-related genes (ACOX, ECH, and CAT) and IDP (Figure 7). Furthermore, genes encoding respiratory chain complex I (NDUFA and NDUFB subunits) and cytochrome c showed significant upregulation. In contrast, at pH 6.0, the mitochondrial gene SOD2 was not downregulated.

#### 3.5.4. Transcriptional Alterations of Amino Acid Metabolism and Nitrogen Assimilation Pathways

Comparing TU546 (pH 5.5) with TU389 (pH 6.0), PLP-dependent transaminases involved in β-alanine, BCAA, and arginine biosynthesis were significantly upregulated. Within the glutamate network, asparagine synthetase (AS) was upregulated, while SSADH was downregulated, corresponding to an accumulation of glutamate (8.28 vs. 6.57 g) and aspartate (5.12 vs. 4.47 g). Additionally, N-acetylglutamate synthase (NAGS) was induced, and arginase (ARG) was repressed. For nitrogen assimilation, glutamine synthetase (GS) and nitrite reductase (NiR, log_2_FC = 1.015) were significantly upregulated at pH 5.5. At pH 6.0, NiR induction was comparatively lower (log_2_FC = 0.47) (Table S6). Finally, phosphoglycerate mutase (PGAM) was downregulated, while acyl-CoA dehydrogenases were upregulated in TU546 at pH 5.5.

#### 3.5.5. Transcriptional Changes in Cell Cycle Progression and Signal Transduction Pathways

At pH 5.5, genes of the cyclin A, B, D, and E families were significantly upregulated in TU546, a trend not observed at pH 6.0. This was accompanied by the synchronous upregulation of Ras guanine nucleotide exchange factors (Ras GEFs), components of the Ras-cAMP-PKA signaling pathway, and various protein kinases, phosphatases, and WD40 domain-containing proteins. Furthermore, the expression of homeobox-domain proteins and rhodanese-like proteins was notably elevated at pH 5.5.

## 4. Discussion

### 4.1. Engineering Strategy and the Role of ADA6 in Carbon Flux Redirection

The heterologous expression of the acylating acetaldehyde dehydrogenase ADA6 from *Buttiauxella sp.* S04-F03 [28] emerged as a highly effective approach for streamlining acetaldehyde catabolism in *C. jadinii*. In yeast, inefficient acetaldehyde conversion often precipitates acetate accumulation and disrupts the intracellular CoA cycle, which significantly restricts carbon utilization efficiency. By utilizing NAD^+^ and CoA as essential cofactors, ADA6 facilitates the irreversible oxidation of acetaldehyde directly into acetyl-CoA. This metabolic bypass not only maintains the homeostasis of the intracellular CoA pool but also fundamentally suppresses the diversion of carbon flux toward acetate byproduct formation [45]. These results indicate that overexpressing ADA6 substantially fortifies the metabolic flux toward the central carbon metabolism (Figure S3). This modification enhancing the strain’s capacity for acetate assimilation, thereby mitigating the growth inhibition typically observed in ethanol–acetate co-fermentation systems.

### 4.2. Impact of pH on Biomass Accumulation and Protein Synthesis

Notably, this study identifies pH 5.5 as the optimal pH for the engineered strain TU546, a departure from the pH 6.0 typically favored by wild-type *C. jadinii*. At pH 5.5, TU546 maintained a robust equilibrium between cell growth and protein synthesis, resulting in high levels of biomass (46.7 ± 0.90 g/L) and protein yield (21.69 ± 0.56 g/L). Although pH 6.0 promoted the highest relative protein content (52.83 ± 1.68%), the accompanying reduction in biomass limited the ceiling for total productivity. This pH-mediated control of carbon flux mirrors observations in other non-conventional yeasts like *Yarrowia lipolytica* [46]. Furthermore, from an industrial bioprocessing perspective, the acidic environment (pH 5.5) also offers a strategic advantage by reducing the risk of microbial contamination, which is essential for large-scale industrial fermentation [47]. Given the divergent optima for growth and protein accumulation, a dynamic pH control strategy, maintaining pH 5.5 during the primary growth phase before shifting to pH 6.0, presents a high-potential approach for further maximizing fermentation performance.

### 4.3. Adaptability to Industrial Substrates and Nutritional Quality

The engineered strain TU546 exhibited remarkable adaptability to a mixture of ethanol and acetate, designed to simulate the composition of industrial off-gas fermentation broth. While acetate typically inhibits industrial yeasts like *S. cerevisiae* by disrupting intracellular pH homeostasis, TU546 maintained the capacity for the simultaneous assimilation of both carbon sources [48]. The near-complete consumption of acetate during 5 L scale fermentation suggests a highly efficient acetate metabolic pathway, providing a competitive edge for industrial biotechnology. From the perspective of food and feed applications, the amino acid profile of TU546 is particularly noteworthy. The essential amino acids, specifically leucine and lysine, reached substantial levels. In comparison with *C. jadinii* CBS 621 cultured on ethanol [16], the engineered strain TU546 demonstrated a 13.3% enhancement in total amino acid content (48.79 g/100 g). Significant improvements were specifically observed in the levels of aspartic and glutamic acids (Table 1), which are essential for nutritional and functional quality. These findings highlight the viability of TU546 as a premium SCP candidate for sustainable animal feed and broader industrial protein sectors.

### 4.4. Metabolic Rewiring and Redox Homeostasis

The physiological superiority of TU546 at pH 5.5 is driven by profound metabolic rewiring. The synchronous upregulation of mADH II, ALDH, and ACS, combined with the significant repression of PDC, indicates the successful establishment of a pyruvate dehydrogenase (PDH) bypass. This reconfiguration effectively minimizes carbon leakage into fermentative byproducts, instead channeling metabolic flux toward the acetyl-CoA pool, a strategy aligned with optimized *S. cerevisiae* models [49]. Beyond carbon partitioning, the activation of the glyoxylate shunt, mediated by the induction of CS, MDH, and ICL, enables the strain to circumvent the decarboxylation steps of the TCA cycle. This bypass reduces CO_2_ evolution and facilitates the preservation of carbon skeletons for downstream amino acid biosynthesis.

The intensification of β-oxidation and C2 substrate assimilation necessitated a robust response to oxidative stress. At pH 5.5, TU546 sustained intracellular balance through the induction of catalase (CAT) for H_2_O_2_ scavenging and the upregulation of IDP to bolster the NADPH supply. Conversely, the sustained expression of SOD2 at pH 6.0 suggests an elevated oxidative burden, potentially triggering a shift toward complete substrate oxidation for energy production at the expense of biomass accumulation.

### 4.5. Nitrogen Assimilation and Cell Proliferation

Environmental pH masterfully regulated both global cellular energy and nitrogen allocation. At the optimal pH 5.5, the stable electrochemical proton gradient (proton motive force), maintained by the plasma membrane H^+^-ATPase, thermodynamically drove the efficient secondary active transport of ammonium (NH_4_^+^) [50]. This nitrogen flux was further supported by the upregulation of GS and NiR [51]. The expanded intracellular nitrogen pool (e.g., glutamine and glutamate) supplied essential macromolecular precursors for cells to pass the G1/S restriction point. This triggered the upregulation of cyclins (A, B, D, and E), accelerating cell cycle transitions [52]. Concurrently, the Ras-cAMP-PKA signaling network acted as a vital nutrient-sensing hub, linking nitrogen availability with carbon signals to globally activate growth-related transcription.

Conversely, at pH 6.0, cells prioritized stress defense over proliferation. Utilizing C2 substrates (acetate) at suboptimal pH exacerbates intracellular acidification via weak acid dissociation. Consequently, cells must divert substantial ATP toward proton extrusion to maintain pH homeostasis [53], restricting resources for biomass synthesis. Mitigating this weak acid stress via precise pH control unleashes *C. jadinii’s* inherent capacity to efficiently assimilate short-chain fatty acids, a trait highly advantageous for sustainable single-cell protein production.

However, while this study establishes a robust metabolic framework, certain limitations must be critically acknowledged. First, the bioprocess was validated at the 5 L scale; potential scale-up challenges related to mass transfer and aeration in large industrial fermenters remain unaddressed. Second, the strain’s robustness requires further evaluation in authentic, unpurified syngas fermentation broth, which may contain complex trace inhibitors not simulated here. Finally, while transcriptomics provided crucial regulatory insights, future studies utilizing multi-omics (e.g., proteomics and metabolomics) are needed to precisely quantify metabolic fluxes. Addressing these limitations through multi-omics-guided engineering and continuous fermentation scale-up will further solidify TU546 as a highly promising chassis for the sustainable industrial production of single-cell protein from C2 substrates.

## 5. Conclusions

*C. jadinii* is a GRAS industrial microorganism, but its wild-type strain shows low efficiency in utilizing ethanol/acetate mixtures. In this study, we engineered the strain TU546 by overexpressing the acylating acetaldehyde dehydrogenase gene *ada6*, which greatly improved C2 substrate utilization and reduced substrate inhibition. After bioprocess optimization at pH 5.5 in a 5 L bioreactor, TU546 exhibited significantly higher biomass (46.7 g/L), protein yield (21.69 g/L), and substrate conversion efficiency (increased by 12.05%) compared with the wild-type strain. Transcriptomic analysis revealed that the high-performance phenotype was attributed to balanced carbon–nitrogen metabolism, including an expanded acetyl-CoA pool, upregulated C2 assimilation genes, activated glyoxylate shunt, enhanced energy supply, and optimized nitrogen assimilation. Future engineering targeting the glyoxylate shunt and amino acid biosynthesis can further relieve metabolic bottlenecks and improve SCP production. This work provides an effective bioprocess for valorizing syngas fermentation broth and lays a solid foundation for the industrial production of SCP from C2 substrates using *C. jadinii.*

## Figures and Tables

**Table 1:** Comparison of amino acid composition between TU546 and *C. jadinii* CBS 621.

Amino Acid	Total Amino Acids (g/100 g)	
	TU546	*C. jadinii* CBS 621 [16]
Essential amino acids (EAAs)		
Isoleucine	2.33	2.36
Leucine	3.64	3.55
Histidine	1.04	1.00
Lysine	3.61	3.68
Methionine	0.68	0.59
Phenylalanine	2.20	2.10
Threonine	2.95	2.39
Tryptophan	0.58	—
Valine	2.77	2.78
Non-essential amino acids (NEAAs)		
Tyrosine	1.95	1.72
Serine	3.12	2.46
Proline	1.81	1.71
Glycine	2.32	2.26
Alanine	3.15	2.91
Glutamic acid	8.28	6.57
Aspartic acid	5.12	4.47
Arginine	2.69	2.49
Cystine	0.55	—
Total amino acids	48.79	43.04

## Data Availability

The original contributions presented in this study are included in the article/Supplementary Materials. Further inquiries can be directed to the corresponding authors.

## Supplementary Materials

The following supporting information can be downloaded at https://www.mdpi.com/article/10.3390/foods15091464/s1. Figure S1: Antibiotic susceptibility test of TU389; Figure S2: Functional verification of the ARS4 replicon; Figure S3: HPLC chromatogram of acetic acid; Figure S4: Transcriptome-based GO and KEGG enrichment plots and volcano plots of differentially expressed genes; Figure S5: Statistical charts of transcriptome functional annotation; Table S1: Plasmids utilized in this experiment;Table S2: Strains used in this study; Table S3: Primers Used in This Experiment;Table S4: Gene sequences utilized in this experiment; Table S5: Statistics of Transcriptome Sequencing Data; Table S6: Names and Abbreviations of Differentially Expressed Genes.
