# Supplementary material for: Enhanced Biomass and Protein Synthesis in Engineered Cyberlindnera jadinii Growing on Ethanol/Acetate: Metabolic Engineering and Transcriptomic Mechanism
Source: Foods. 2026 Apr 22;15(9):1464. doi: 10.3390/foods15091464 (PMC13163513; doi:10.3390/foods15091464)
Supplement: Supplementary file 1 [file foods-15-01464-s001.zip › foods-4240300-supplementary.pdf]

Supplementary Material

List of Supplemental Figures

Figure S1. Antibiotic susceptibility test of TU389
Figure S2. Functional verification of the ARS4 replicon.
Figure S3. HPLC chromatogram of acetic acid
Figure S4. Transcriptome-based GO and KEGG enrichment plots and volcano plots of differentially expressed genes
Figure S5. Statistical charts of transcriptome functional annotation

List of Supplemental Tables

Table S1. Plasmids utilized in this experiment
Table S2. Strains used in this study
Table S3. Primers Used in This Experiment
Table S4. Gene sequences utilized in this experiment
Table S5. Statistics of Transcriptome Sequencing Data
Table S6. Names and Abbreviations of Differentially Expressed Genes

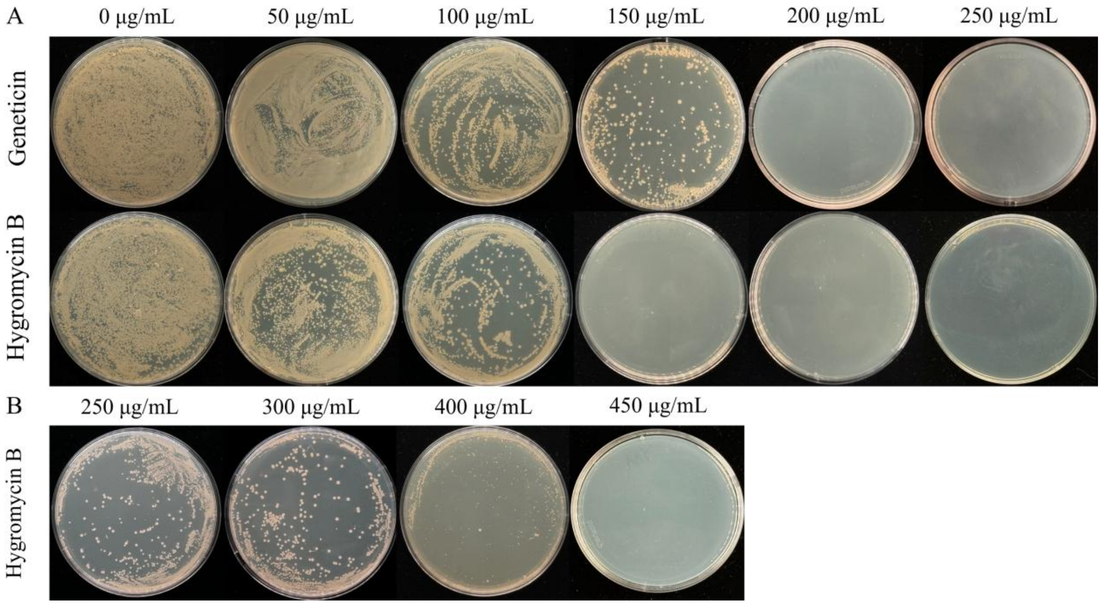

Figure S1 Antibiotic susceptibility test of TU389

(A) Growth of wild-type TU389 at different antibiotic concentrations. (B) Sensitivity of electroporated TU389 to different concentrations of antibiotics.

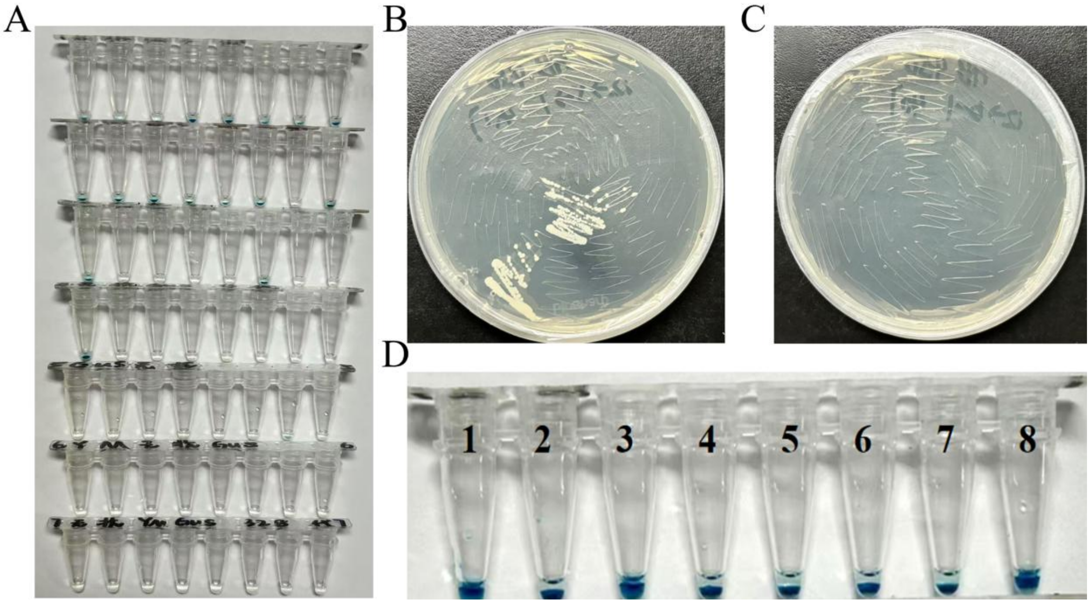

Figure S2. Functional verification of the ARS4 replicon.
(A) GUS staining verification results of Hyg-ARS4 plasmids after 1–7 consecutive passages under antibiotic-free conditions. (B) Verification results of the 5th generation transformants from antibiotic-free plates streaked onto antibiotic-containing plates. (C) Verification results of the 6th generation transformants from antibiotic-free plates streaked onto antibiotic-containing plates. (D) GUS staining verification results of the 5th generation transformants from antibiotic-free plates after streaking onto antibiotic-containing plates.

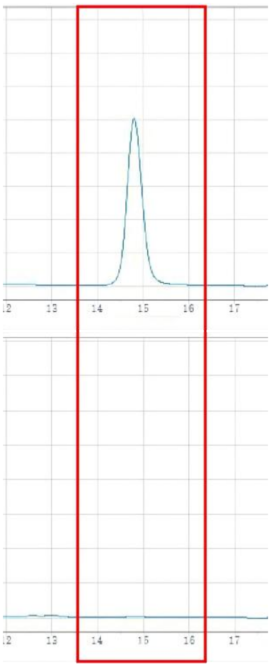

Figure S3 HPLC chromatogram of acetic acid

Note: The retention time of acetic acid is 14-15 min. The lower part of the figure shows the HPLC detection result of acetic acid in engineered strain TU546 at the end of fermentation, and almost no acetic acid was detected.

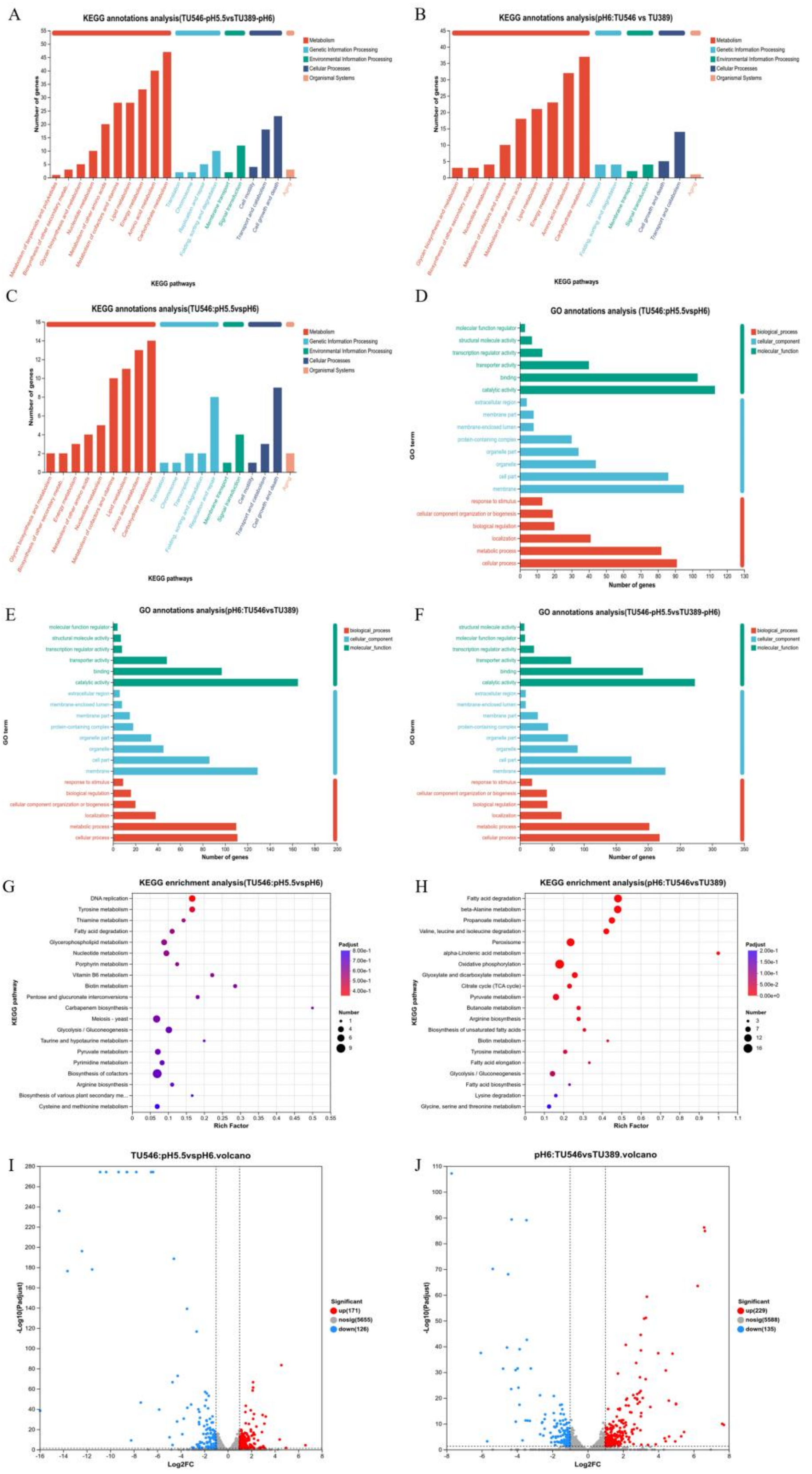

Figure S4 Transcriptome-based GO and KEGG enrichment plots and volcano plots of differentially expressed genes

A: KEGG annotation plot for TU546-pH5.5 vs TU389-pH6;

B: KEGG annotation plot for TU546 vs TU389 at pH 6;
C: KEGG annotation plot for TU546 at pH 5.5 vs pH 6;
D: GO annotation plot for TU546-pH5.5 vs TU389-pH6;
E: GO annotation plot for TU546 vs TU389 at pH 6;
F: GO annotation plot for TU546 at pH 5.5 vs pH 6;
G: KEGG enrichment bubble plot for TU546 at pH 5.5 vs pH 6; Volcano plot of differentially expressed genes (DEGs);
H: KEGG enrichment bubble plot for TU546 vs TU389 at pH 6;
I: Volcano plot of DEGs for TU546 at pH 5.5 vs pH 6;
J: Volcano plot of DEGs for TU546 vs TU389 at pH 6.

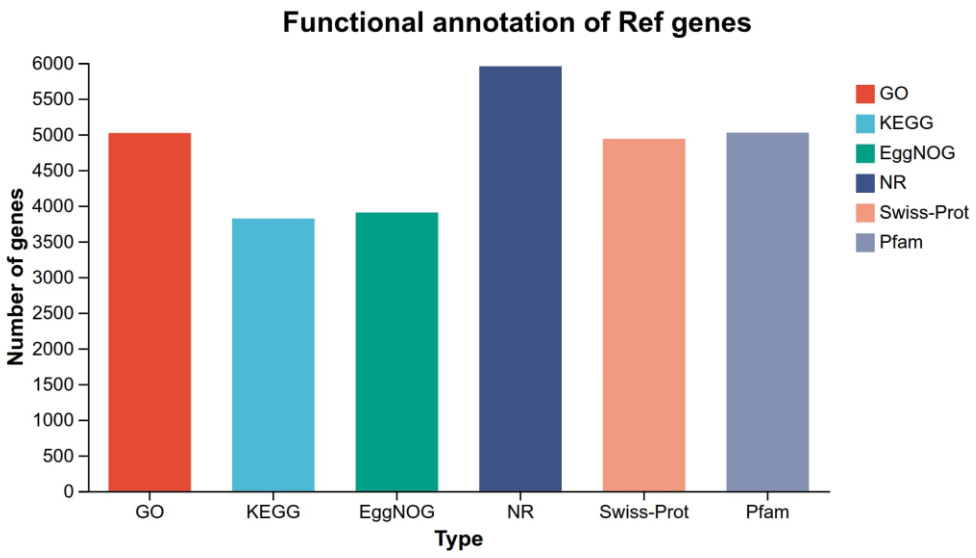

Figure S5 Statistical charts of transcriptome functional annotation

Table S1 Plasmids utilized in this experiment

No.	Plasmid name
1	PMD18
2	pMD18-Hyg
3	pMD18-Hyg-ARS1
4	pMD18-Hyg-ARS2
5	pMD18-Hyg-ARS3
6	pMD18-Hyg-ARS4
7	pMD18-Hyg-ARS4-GUS
8	pMD18-Hyg-ARS4-GUS-P_{GAPDH}
9	pMD18-Hyg-ARS4-GUS-P_{PGK}
10	pMD18-Hyg-ARS4-GUS-P_{PGK}-ADA6

Table S2 Strains used in this study

Designation	Strains
HygB-ARS1	*C. jadinii*-*HygB*-ARS1
HygB-ARS2	*C. jadinii*-*HygB*-ARS2
HygB-ARS3	*C. jadinii*-*HygB*-ARS3
HygB-ARS4	*C. jadinii*-*HygB*-ARS4
GUS-HygB	*C. jadinii*-*HygB*-ARS4-GUS
P_{PGK}-ADA6	*C. jadinii*-*HygB*-ARS4-GUS-P_{PGK}-ADA6
P_{GAPDH}-ADA6	*C. jadinii*-*HygB*-ARS4-GUS-P_{GAPDH}-ADA6
P1-P56	*C. jadinii*-ARS4-GUS-P_{PGK}-ADA6 (ApaI-linearized)

Table S3 Primers Used in This Experiment

Primer name	Primer sequence
ARS1-F	gtcgactcgtcctttcctac
ARS1-R	aatgcgttatttaaataaagatccactagt
ARS2-F	gaattccaaactaatttgaaaagctca
ARS2-R	caatagtagagcgcataatgca
ARS3-F	ataaaaacacaacaaagctatgaaagc
ARS3-R	ggctatgtcactttaagttgttgag
ARS4-F	gatcatgtttcgtacaaaacaccgc
ARS4-R	aatcttcgcggaattgagataagatc
Pro-F	gagctcggtacccggggatccccacatttgttgtacgagaagggc
Pro-R	gctttttcataccgttgttctctctttggaaaacg
Hyg-F	tccaaagagagaacaacggtatgaaaaagcctgaactcaccgc
Hyg-R	ctcgatcgattctatttctttgccctcggacgag
CYCI-F	aaagaaatagaatcgatcgaaaatttaaagaaaaccatactatacacataca
CYCI-R	gacgatatctctagaggatccctataggggcgaattgggtaccg
P_{GAPDH}-F	agctcggtacccggggatccaagcttacagcgagcactca
P_{GAPDH}-R	gggtttctacaggacgtaacattatgttgtttgtaagtgtgttttgtatctgttg
P_{PGK}-F	gctcggtacccggggatccaagcttttgtcttttaggagccttct
P_{PGK}-R	ggtttctacaggacgtaacatctttatccgccagtatgttagtccaa
GUS-F	gaacaacggtatgttacgtcctgtagaaaccccaac
GUS-R	gaggctgtagccgacgatggtgcgccaggagagttgttgattcatgtttgcctccctgctgcgg
ADA6-F	taacatactggcggataaagatggatagacaacaaatcgagcaag
ADA6-R	ctttaaattttcgatcgattttaaacgattctgaaaccatcaaccaaaac
T-P_{PGK}-F	gcggaattgagataagatcgggcccaagcttttgtcttttaggagcctt(including ApaI)
T-P_{PGK}-R	tcgatttgttgtctatccatctttatccgccagtatgttagtcca
T-P_{GAPDH}-F	gcggaattgagataagatcgggcccaagcttacagcgagcactcaaatc
T-P_{GAPDH}-R	gctcgatttgttgtctatccattatgttgtttgtaagtgtgttttgtatctgttgg
T-ADA6-F	taacatactggcggataaagatggatagacaacaaatcgagcaag
T-ADA6-F	ctttaaattttcgatcgattttaaacgattctgaaaccatcaaccaaaac

Primer name	Primer sequence
T-CYCI-F	ggttgatggtttcagaatcgtttaaaatcgatcgaaaatttaaagaaaaccatactatatacacatac
T-CYCI-R	ttctcgtacaacaaatgtggggatccccgggctataggggcgaattgggta(including ApaI)

Table S4 Gene sequences utilized in this experiment

Gene Name	Gene sequence
HygB	atgaaaaagcctgaactcaccgcgacgtctgtcgagaagtttctgatcgaaaagttcgacagcgtctccgacctgatgcagct<br>ctcggagggcgaagaatctcgtgctttcagcttcgatgtaggagggcgtggatatgtcctgcgggtaaatagctgcgccgat<br>ggtttctacaaagatcgttatgtttatcggcactttgcatcggccgcgctcccgattccggaagtgcttgacattggggagtttag<br>cgagagcctgacctattgcatctcccgccgttcacagggtgtcacgttgcaagacctgcctgaaaccgaactgcccgctgttc<br>tacaaccggtcgcggaggctatggatgcgatcgctgcggccgatcttagccagacgagcgggttcggcccattcggaccg<br>caaggaatcggtcaatacactacatggcgtgatttcatatgcgcgattgctgatccccatgtgtatcactggcaaactgtgatg<br>gacgacaccgtcagtgcgtccgtcgcgcaggctctcgatgagctgatgctttgggccgaggactgccccgaagtccggca<br>cctcgtgcacgcggatttcggctccaacaatgtcctgacggacaatggccgcataacagcggtcattgactggagcgaggc<br>gatgttcggggattcccaatacgaggtcgccaacatcttcttctggaggccgtggttggcttgtatggagcagcagacgcgct<br>acttcgagcggaggcatccggagcttgcaggatcgccacgactccgggcgtatatgctccgcattggtcttgaccaactctat<br>cagagcttggttgacggcaatttcgatgatgcagcttgggcgcagggtcgatgcgacgcaatcgtccgatccggagccggg<br>actgtcgggcgtacacaaatcgcccgcagaagcgcggccgtctggaccgatggctgtgtagaagtactcgccgatagtgg<br>aaaccgacgccccagcactcgtccgagggcaaagaaatag
Pro	ccacatttgttgtacgagaagggccaacgacagctctcttcgaggaagacttggagataagccttgcttgatttgtaatcttcaa<br>gagagagctctttgtagcttgtcttgttgagtgtctgagaagcatttgtgcaatgaatatgggagagatgagatgagtagagag<br>cagcacaagtggaatcaaatcacaataacaactttagccacagggaggttaaaagaggagaagaaggagtcctttccaattg<br>tggctagtgcagaagagaaaatttgcttgctacaatcgtggtgtgtatgcaaacccgttgtaaaggtggtgtctttgtatatgtag<br>ggtgtgtggcttcgtctcgagaaagcacataagctgtggcgcactttctcgggtaagtgatttaattgcacgtgatctcaatttct<br>ttttttgaagccactaaagcttacgtaagcgaccacggatctggtgttgggatgttttggttttgggaggggcaggggggtttaca<br>tgttggctttatcgattgcggcgctttgtgtttgggggtgtatgccctagcgaccctgtgggccactgcccaggtgcccaggtg<br>cgaccaggaaaaaaatttcttcatcgctagagctttcttcaaccccctttctttcctaattcttttcaactaacaacaaataaacaca<br>gtaacaagatgtcatctgacctttcagacgttagactctttgtcagaccacttccattcgatgttaacgaagaggacttgaagag<br>cttcttctcccctattggtgaaatcaccgatttcatcgttgctagaggttatgcctttgttgaatacgctaatgcagatttggcaaga<br>caagccatcgctgaattgcaccaaaagccgttcggtgatgttccattatccttggagtacgctaaggctcaaaagccaagattc<br>agacttcttgtttctaacatgccagaaggtgctgagtggcaagatctgaaagattttgctctccaaaagggattcgaagttacct<br>acgccaacgttttccaaagagagaacaacggt
ARS1	gtcgactcgtcctttcctactggttgtagcagctagtaagggttagactgttcgtctattaaaagggtacgtgagttgggtcttata<br>tttggcccctttacattgtaaaatgtattgaaaaataggtttaaaacggtgaacgctaagttaattaattaattaattaatatgtgaat<br>accgtgggaattaataatattaataaaaatattgattaacccgtaacgactcaattttaaataaattggatacataatagttaagtat<br>ttaaacttaatgaaaacactttatgtataatcagacctatatctttataattaaaacagtaatatatattacatgtaagtataagtatta<br>gttaattataataactaatataattgattaaaaaatatataatgaataaaaatacaataaataaacaaaaaataaatatgaattatga

ttatttatctggaatatttcatgcagatggaacatttacagtatcaataattaaaggtaaaaataaattatatttgaatcctcgtatgat
ttttacacaacatgagagatcattaaatattattaaagaaatgaaattaaaatttaataatgttggacatattaaatttcaaacaaata
aaatttgtaaatatacaattacaaatcttgaagatataaaaaaatatgttttaccagtatttgataaatatactgtaagaagtaataaa
tattcaggttatttaaaatataaattaattgttaatattttatattttgagaaaccaatatataattcttctttatgatgattttgtttttatttat
caactaaaattaatcctttagttaaaataagtaaacaaatgcgttatttaaataaagatccactagtgggccc

ARS2

gaattccaaactaatttgaaaagctcaatttctttgaaatattttctgaaatattttggtcctacttattctttgctttttttctattaaggtc
cattgatcttgagaactgcttattcaaacctagctattatcggcctttgtattgaaaaggaataccttgtcatagaatgtagtttttttt
tgatttttctttttcgaagacagcagcttctcaatttatcctcactttgaattgagttccagtttattatgtccacaataaaattgcacg
accagaaatccaagatcataaaacaaaattgaatcatacatcggaacctggcaaaaaatagaggcaacctaatgaactgata
caaacaatcaaataaatcaagtctgctctctcaatataaataaaaagaaaagttactctatatcagagttttcgatcattgagcaa
gaaaactataattaaaggatttgtatgtaatcaattggcaaccagaagcttttaatagtgattgtagacagaaatttggaatgatg
gtttgaacataaaatgtgcgagatggaaaacctgattctaaacatcccaggttgctatgtttaagcgccaatggggttttcgcag
ttattttaagaatacttcgtaattgtttgtacttttattacctcgaaagtattatatataaatgaaggcactgtttaccaaaaaaataaat
tttattaggatgagtgttgtcttgtaatttgtttttttaaaagtgcaacagcatatgaggatgaagaaagtaatacgttattattggcat
atcataattcgtgatggattaggatgttttaccgctctgaaagctaaatatgtttgagtgaaagattaatcaatagggaaccatcgt
tgtcatgaaatagtgaaagtgcgagtaatatgaaaaccaatattaagaacatttcttttaggatttaaaaataatttagcatttttatg
tgggtttggtgattctgaattaaagttgtctgtaatgaagtaaattcgaatagtatattgggatcactttgtatatttgggacactgg
gttacgtaggtcatcttttctctgattagatgaccaacatttcaagctgagagccagttttgtaacattaatgaatttttttaatgtgga
cttactcagacctgcaatctgttttcaatattttgtcgtgtttccctataaagttatacgcaatatttattaaggatacgaacaaggtg
agttttgttttctatatcatggaaaccataggtggactatctttaataaaaaagaggcatctaaatagctgagcaaagataacaat
aaaatagtatcaagggaaaagatggttgaaataaagtaatttattttgtagttaattgcgaagaattaaagatattggtattttgaa
aacgaaaaacaaaatcaaaaaaagactttagtggaatttgtttatccgtaagctattttcaatagtagagcgcataatgcagggc
cc

ARS3

aagcttatagaatttgaagatataaaaacacaacaaagctatgaaagcaatgaggggacgatttgatgaacagaaggagcta
ttcccattaatttaatatctaccaatagatatgtcagtcaaacatgtcagaagtcatactcactaattatatgagcgggtgcgttag
atatttatatgagtttgcatctttctacatggggctttcaagtaactggaagtaatacaacttttgtttaagttgataaaaacaaaaaa
caaaaaaacaaaaaacaaaaaaacaaaaataaaaaaaaaaaaaaacaaaaaaaaaaaaacacacacgcacacacacaca
tatacaaacacatacaaaaaacttatcataattaagataaatgaaagctatctaaaatttccagacattttctgaaaaagtggctg
ccagctttattgctttgctttaaatttataaagaaaacttctttgaaatcgaatatgaaacaagaggaaacggatgaaaggataaa
acacaaatacaggaaaacattattacaaataaagcacctgtaagagataaatttgttacatttaaaggattctacttacatataca
gagaaagcaatttcatagacatagggtctaccgaacagtcttgatatttcagactagtatttttgttgtattatggggcttggtcgg
tatgttagtaaaaagttcattttaaaattttccaagaagtgtttttattgcagaaaaatatccgtggttcaagagataatgggctgta
aatttgttttgtaccaaaaatatcttaattaatacaaagaatacctttatgaaggtagatcaagatcttaaatttcattactcagaaat
gaatatacttgaaacttccgaaatactatgttatggggaacaaataagaggagccatttcatatttattttggaaagatcgttttcta

tgcgcagttgttggaatagcgatattatcatgaccttatattcagtcagagaaaatagggtacgaatttgaaaacaatgtttcagc
ttcaaagaggacctttaaacggtcaggcaaaagttgaggtgtcagtgtgtataaaaatgttcaattcatttttggttgaaagatgc
tttaaaaggttggtgcaaagaatcatatatgtgtattggctagttaaaagttgctttattaaaaatatatgcaaactaaattgtctata
cgattgataaggtgaaacttagataaacaatgaaaaaggaaggtgctttgaaaaccgaccagcttcaaataaatatgtaactat
ttttatggatgtgaaaattaaatgttgtcgaatctgctgtttctagatttgtagatgaaaatgttgacgtgagagttttcatttgtttgta
ttttatattatgctttgattactactcatagcttgggtttagcatggcctgagtaagtaggaagatccaataaattgactgttgtcgttt
tgaaattaaatactgaaatgaataaaagtttgacgagaaaagacctgaaatatataaaaatgttttgtattatttaagtcggttacat
tctctcactttattgtaacaaccattatagtgatggggaaaaaataaaacataagccacataaggagatattgttctttattgaaag
gatggaatcattttctggaaatgtcaaaaattaaatattacttggtttttgatgaattgtagaagaaaaagtaaatgctgctattctct
ttctttacattttccatgtttcctgattctggctatgtcactttaagttgttgagatatcgggccc

gatcatgtttcgtacaaaacaccgccactccgttatgagaaagtcatagttatatttcggggaaacttatgttgcttgcaaggaat
aatagacagacaaatgttttacgaaactgaaggattaatacaattgatgcaaaaaaacaaaaaaaaaaaaaaaaaaaaaaaa
aaaaaaaaaacaaaaacacaaaaacacaaagaaaaaaaacacaaagaaaaaaaacacagaacatccaaaacaaaacaac
aatatatatatatttgctaataccatcaccctccctcatacaaaaaaaaaagaaaatggaaggagcacgtatatcttttctatgatc
tttggatatggaatagataagcaacccctctagggaagtacacatcaagatacttggggagcaaactgagagcacatgatata
cacgaaagccaccatatatcatatataaaaatatgaaacatgaaaagttattcatctgttggattcacttttatatgttttcatgcatt
gtctactctatgcctctgtcttttctctgttcctttacactcctctattatcaaattgagtgtttcatttatcaaagaagtttgagctggat
cagaactttagatattcattcctgttttcgatatatctatacgatgttctaatatccactctcactaccattgttaaaaaaagttaaaatt
atagctgtgtgccttaagggaataaaggaaatggatcttttgaatgttaaaaaacgagatactcttttgtaaaccagaaaacgatt
ttcaaaacacaaattggtgaatgtcaccaagcaaaaattgtatcctaaaaaaaataaatttatgaactaaattatctctgaacaga
catttagtcaaccttttctccttgctcctcggtcaaaggtttttcgtatagatatatatacgggttgctttttgtttccactcgtctaatc
ARS4 gagtttcgatatcaatggagatttattctttggctttgattccataataatccatatcctaataaaacactttgaagcgaattgaaac
cccaatatctttctggccattaaaacatttataaagtactggatgtttaaagagctttgagaattgcctagcttcaaaatatatttgtc
tccaattatgattttgtattttcctttctttgttttctgtagttatttaaaataagttcactacgttgtttttgaggaaccgttactctattta
ctcaaatttattatcaaaatgtttttttttcgtttgatttattcaaatgctgtcgatatgtcccagaaatatcatacaattcaaatttctaa
agccagcgtttattataaagctttgagttctttcgacttaattacatgtatgtagctcaaaccaaagttactctataattataaaaaga
ctatgaaccaattcaagaattccccatttccagcaaatttagtatagctcaaattcacactgtcatatgcaaaaacctaaataagc
agatcattgtaaagagccggcagttgtatattccagtggggctgaacttgtggttaggattcacagacatcttgttgtcggatttc
tattgatagaagctgtgccattgaaaatggaaatataaaatggtattgggttgatcatatatgaatttcttattactcataataatag
gagaaatcatcgaacatggaacatagatgctaattaaggtacgtacagcatcctgttcaatatttcaactttttaagtattaaatta
gtgaagaaatgtattatgaaccattgttcaataattctaatgtgtttttgtggtttttttttggcttttgggacattgtaatttttactcat
ttattcgatgtctcttcaggtttttgtgttttttttttttgtgtttaaatcttcgcggaattgagataagatc

ADA6
atggatagacaacaaatcgagcaagttgttaaggctgttttggctggtatggctgctaactctgctcctgagcctgttaccccac
catgtggcaccggtgttttcgcttctttggatgatgctgttcaagctgcttctgttgctcaaaaggctttgacctctgttgctatgag

acaaaaggttgttgctgctatcagattggctggtgagcaacacgctcaacaattggctgagatggctgttgctgagaccggtat
gggtagagttgctgataagtgtgctaagaacatcgctcaagctagaggcacccctggtgttgagtgtttgaccccacaagtttt
gaccggtgataacggtttgaccttgatcgagaacgctccatggggtgttgttgcttctgttaccccatctaccaaccctgctgct
accgttatcaacaacgctatctctatgatctctgctggtaactctgttgttttcgctccacaccctgctgccaagaaggtttctcaa
caaaccatcacctggttgaacgaggctatcgttgctgctggtggtcctgctaacttgttggttaccgttttgaaccctgatatcga
gaccgctcaaagattgttcaagtaccctggtatcggtttgttggttgttaccggtggtgaggctgttgttgatgctgctagaaagc
acaccaacaagagattgatcgctgctggtgctggtaacccacctgttgtcgttgatgagaccgctgatatcccaagagctgct
aaggctatcgttcaaggtgcttctttcgataacaacatcatctgtgctgatgagaaggttttgatcgttgttgagtctgttgctgatg
ctttgttggaggagatgcaaagaaaccacgctgttttgttgtctgatgctcaagctgagaagttgttgcctgttttgttgaagaac
gttgatgagcaaggtaagggtcaagtttgtagagattgggttggtagagatgctaccaagatcgctgccgctatcgatttgaac
gttgcttctgagactagattgctgttggttcaaaccaccgctagacacccattcgctgttaccgagatgatgatgcctgttttgcc
aatgatcagagttgctaacgtttctgaggctatcgctttggctgttaagttggagggtggttgtcaccacaccgctgctatgcact
ctagaaacatcgataacatgaacgctatggctaacgctatcgatacctctatcttcgttaagaacggtccatgtatcgctggtttg
ggtttgggtggtgagggttggacctctatgaccatcaccaccccaaccggtgagggtgttacctctgctagaaccttcgttaga
ttgagaagatgtgttttggttgatggtttcagaatcgtttaa

aagcttacagcgagcactcaaatctgccctccgagccctccggccctctcttcaacaaactcgcgctgcacttcgtcgtcagt
ggtgccaatcacccaacgtggaggtatcaagaggtgctccagcccacaaagcgacatcaaagacaacaaccctgccggc
ctacgtcctacacaccctggtgatcgcagacattgtacaaggtgccacgcaataacctacaggcaccgcacatgacgatgg
ccttggttgtgcaaccagtgacttccacggtccacgcagcaacatgaaccacaccacccagaatcgatgcgcgcaacaaca
gttgttccggttcactcagccccacagcgagtcgctggcagaacacgagcctgagggcggaaagagggtagaggaaagc
gcaaggacaggggacaacctggcccaattgatgtcatataaaccctctcgatcaattgagcacactcatccgccaattgacc
P_{GAPDH} cctgttcgcagctccacgccccatgttcctcgtccctggtgtagcttctcccctaaattccagcgcttggttccgccctccctgtc
tcccgggtttaacgaacgtgtgtaccatctgatggtaatccgctcccgtccgcgcaacacaactcacaagcagatcacacctg
tacacgccgctgctgatgcgcccaatttaattttttttctctcaatgtaggggagaagccttgggagctcccgactcccagttgg
gcacagctgccacctcatgacttttcctgtgtgtgcctgtctgacgttacgtgtgatgtagtggcccccgttcggtgtgttttcgc
ctgttgcgctgtgcccccccttaaaagtataaaaggaagtgcaattgctgtttgtgttgattgttgatccttgtttcctctgtttcctcct
catcacacaagaaaggtttcttctttccaacagatacaaaacacacttacaaacaacata

aagcttttgtcttttaggagccttcttttcaccctggctttcttcagactccacgcctctcgcccgtttgttgttgatcttcttctgttgc
ttcttcgtgagcttaccagtatccagatgcgttgtcagggcaagagggtcatgttcaagctcctctttcactttcagtccaatacgt
ttccaggcagggatgtgttcgctcatcgttccagactcgagtggtgaaaactatggcaacctctacttcctttccaaacacaca
P_{PGK} gcgtgctttgtagtgtgtgcctaagagctgaattttttttccttccatgctgcgctgcgatgagctctgcccgcccgcagcctcgg
aggctagcgacgtataaaaaaggcctgtgaaaattttatcctcctccttaacgacccttctttctcttcttcacattcaaaaacttca
agcagctgtctctgttcctttgctgtgttctaccacgggacattcccattccccgtggagaaccgaactggagtctagcagcat
gcgagatcaatattacacggtttgagtctgatacgcttgagcagccatttttggcttctcctggtgtgtatccagatatagaagtt

cgtatacatttcccatagcgattgtaaaatgattctgcaatggaacaatccgtaattgtaggcctggtgagatggcactcgcaat
gcctctgtgtgtagtttttttcccttctccgtccatcagcaacagtggcttcttagggcataacgagacggctccttggtgaaaga
tgccctgctccgtctgtctgcctgttgctacaaccactgcgtagtcagatgacccggtctgtgtgctgtggaatcaccgggagc
gaaattccggtttcgctggcagatgagctcatcaaccacatcaactggagcaacctcaccagaggacacgtaacctgcccg
gttgaattctgtcaaaccgtacatcacacaacaacagcaacaacaacaacaacgtcagttgtcgttcgcatgacgacgttacg
taacggcaccaacaccgtatcgtcctcgccaatgcctgtttcccctacccggagtggcccggcccacctgtcgttcttttttcgt
caattgtgtccagctggtgccatcaccatatgttcaagggcgtgacctgtactagcgcagtctgctgcagtataaaagggattg
ctgaggccccctttagcgtttccaatcaacaattgattccttttccccatagtccgtttgtactacatcctacataacaaaagtga
gtgttacaagacaagtgtggcggtcaattggatcatttggactaacatactggcggataaag

Table S5 Statistics of Transcriptome Sequencing Data

Sample	Clean reads	Clean bases	Error rate(%)	Q20(%)	Q30(%)	GC content(%)
TU546-pH6-1	50525684	7600391776	0.0117	99.37	96.23	47.15
TU546-pH6-2	45761908	6881398555	0.0118	99.36	96.18	47.13
TU546-pH6-3	40519976	6090944556	0.0118	99.33	96.07	47.02
TU546-pH5.5-1	40421624	6071541429	0.0118	99.33	96.09	47.21
TU546-pH5.5-2	43514908	6541244864	0.0119	99.33	96.02	47.21
TU546-pH5.5-3	38523972	5785153262	0.0119	99.31	95.96	47.23
TU389-pH6-1	44638492	6714034573	0.0119	99.3	95.88	46.84
TU389-pH6-2	45064220	6765004968	0.0118	99.36	96.14	46.85
TU389-pH6-3	45986322	6911528277	0.0118	99.33	96.07	46.98

Table S6 Names and Abbreviations of Differentially Expressed Genes

Gene Name	Abbreviation	Log2FC (TU546-pH5.5vsTU389-pH6)	Log2FC (TU546:pH5.5vspH6)	Log2FC (PH6:TU389 vsTU546)
Aldehyde dehydrogenase	ALDH	5.67	-1.45	-3.97
Mitochondrial aldehyde dehydrogenase	mALDH	-4.12	-0.42	-3.69
Acetyl-CoA synthetase 1	ACS1	6.43	0.98	5.45
Acetyl-CoA synthetase-like protein	ACS-like	4.96	0.97	3.99
Alcohol dehydrogenase	ADH	-5.42	-1.45	-3.97
Alcohol dehydrogenase 1	ADH1	4.51	0.97	3.54
Alcohol dehydrogenase 4	ADH4	-5.09	-1.87	-3.22
Mitochondrial alcohol dehydrogenase II	mADH II	7.71	1.13	6.59
Pyruvate decarboxylase	PDC	-2.26	-1.48	-0.78
Citrate synthase	CS	1.14	-0.21	1.35
Malate dehydrogenase	MDH	1.60	-0.03	1.63
NADP-dependent isocitrate dehydrogenase	IDP	1.06	-0.29	1.35
Isocitrate lyase	ICL	1.78	0.57	1.20
Acyl-CoA dehydrogenase	ACAD	3.37	0.57	2.80
Acyl-CoA oxidase	ACOX	3.83	0.80	3.03
Acyl-CoA oxidase 3	ACOX3	2.53	-0.51	3.03
Enoyl-CoA hydratase	ECH	1.47	-0.27	1.75
Thiolase	THL	1.61	-0.21	1.82
Catalase	CAT	3.12	0.17	2.95
Mitochondrial superoxide dismutase	SOD2	-10.82	-10.87	0.05
Respiratory Complex I	Complex I	2.12	0.47	1.64
NADH dehydrogenase subunit A	NDUFA	1.50	0.62	0.88
NADH dehydrogenase subunit B	NDUFB	2.09	0.78	1.31
Cytochrome c	Cyt c	1.30	1.35	-0.04
Adenylate kinase	AK	1.47	0.68	0.79
Pyridoxal phosphate-dependent aminotransferase	PLP-AT	2.58	1.22	1.36
Succinic semialdehyde dehydrogenase	SSADH	-1.23	-1.00	-0.23
N-acetylglutamate synthase	NAGS	1.14	-0.21	1.35
Arginase	ARG	-1.78	-0.40	-1.38
Ornithine aminotransferase	AOAT	-4.91	-4.32	-0.59
Nitrite reductase	NiR	1.02	0.55	0.47
Phosphoglycerate mutase	PGAM	-1.64	0.04	-1.68
Adenine phosphoribosyltransferase	AMPT	-1.09	-0.67	-0.42
Cyclin	Cyclin	3.02	2.05	0.98
Ras guanine nucleotide exchange factor	Ras GEF	1.35	0.80	0.55
Protein kinase	—	1.35	1.31	0.04
Protein phosphatase	—	1.94	1.25	0.69
WD40 repeat protein	—	1.98	0.79	1.20

Gene Name	Abbreviation	Log2FC (TU546-pH5.5vsTU389-pH6)	Log2FC (TU546:pH5.5vspH6)	Log2FC (PH6:TU389 vsTU546)
Homeobox domain protein	—	2.55	2.79	-0.23
Rhodanese-like protein	—	1.94	1.47	0.46
